# Supplementary figures and images for: Endothelial SIRPα signaling controls VE-cadherin endocytosis for thymic homing of progenitor cells
Source: eLife. 2022 May 5;11:e69219. doi: 10.7554/eLife.69219 (PMC9071265; doi:10.7554/eLife.69219)

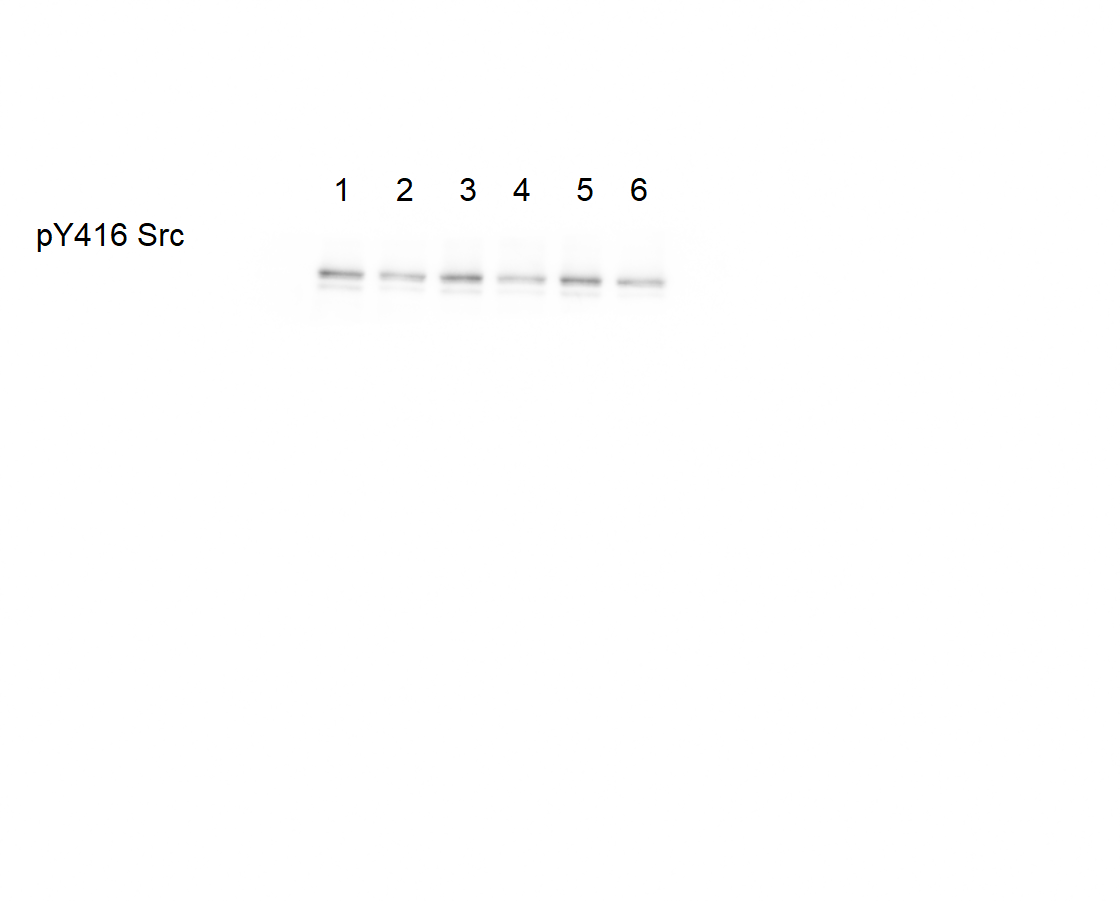

Supplement: Figure 5—figure supplement 1—source data 1. [file elife-69219-fig5-figsupp1-data1.zip › Figure 5-WesternBlot-source data/Figure 5-WesternBlot-source data/Fig.5.D.1.pY416-Src_labeled.tif]

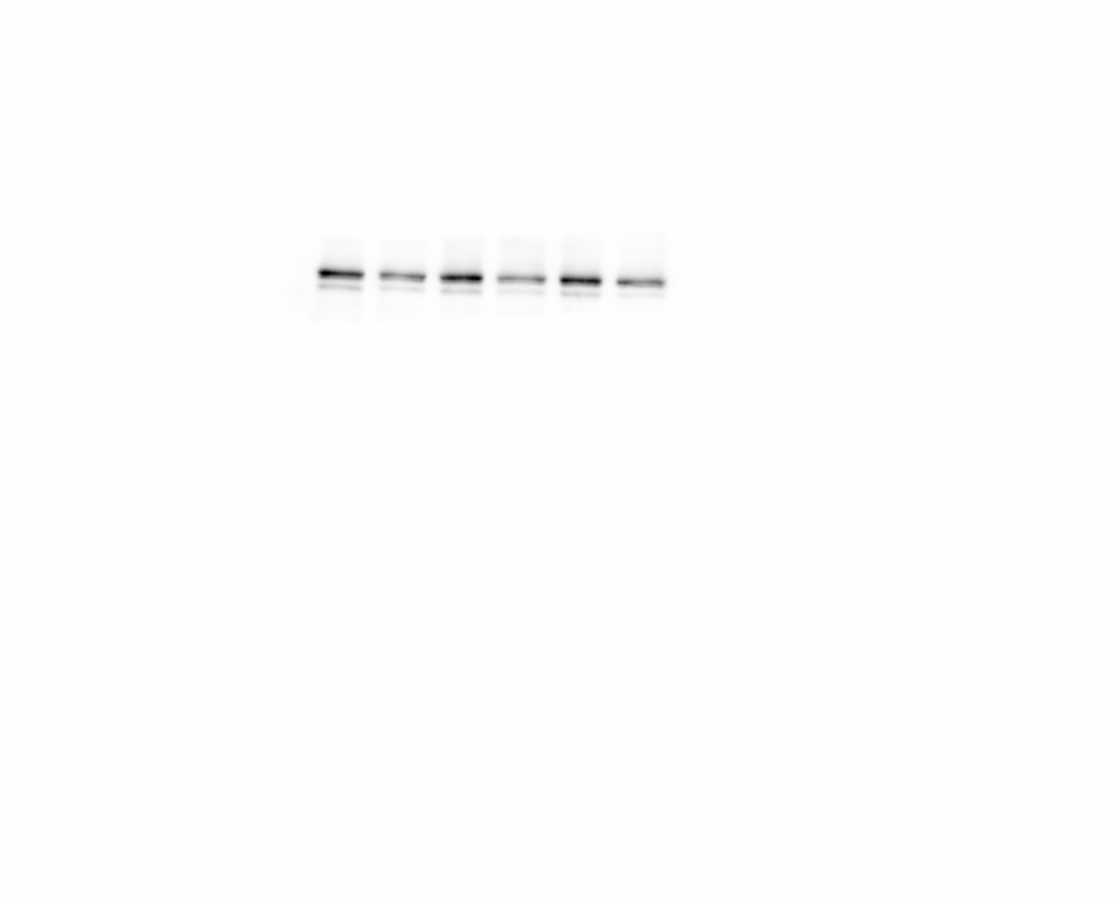

Supplement: Figure 5—figure supplement 1—source data 1. [file elife-69219-fig5-figsupp1-data1.zip › Figure 5-WesternBlot-source data/Figure 5-WesternBlot-source data/Fig.5.D.1.pY416-Src_raw.tif]

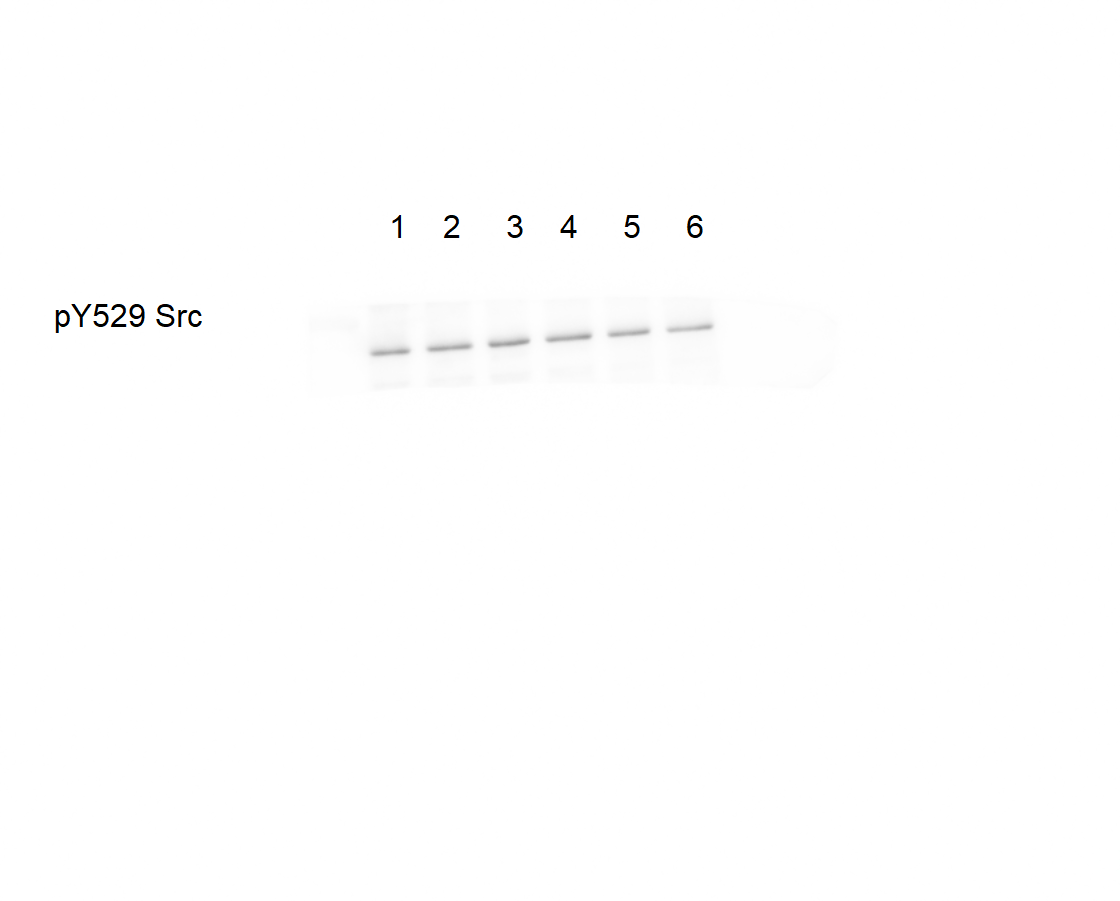

Supplement: Figure 5—figure supplement 1—source data 1. [file elife-69219-fig5-figsupp1-data1.zip › Figure 5-WesternBlot-source data/Figure 5-WesternBlot-source data/Fig.5.D.2.pY529-Src_labeled.tif]

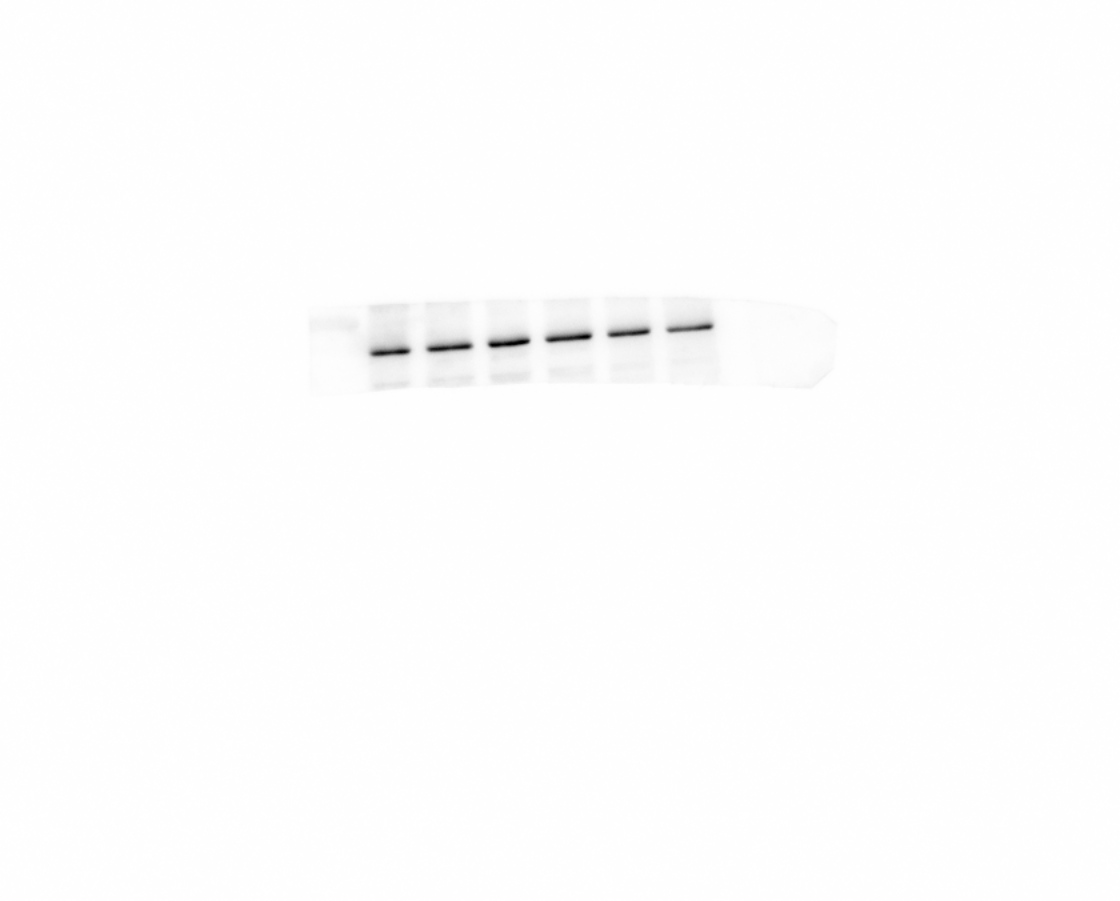

Supplement: Figure 5—figure supplement 1—source data 1. [file elife-69219-fig5-figsupp1-data1.zip › Figure 5-WesternBlot-source data/Figure 5-WesternBlot-source data/Fig.5.D.2.pY529-Src_raw.tif]

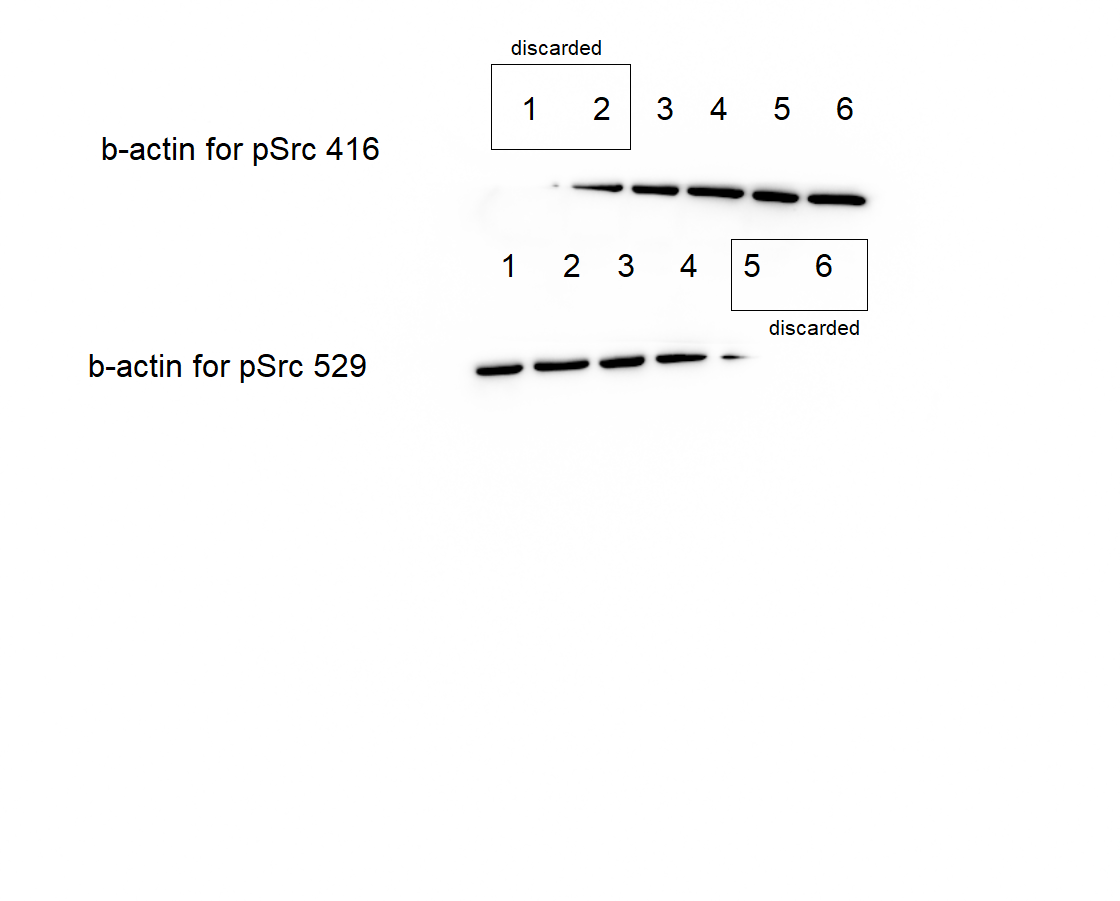

Supplement: Figure 5—figure supplement 1—source data 1. [file elife-69219-fig5-figsupp1-data1.zip › Figure 5-WesternBlot-source data/Figure 5-WesternBlot-source data/Fig.5.D.3-BetaActinx2_labeled.tif]

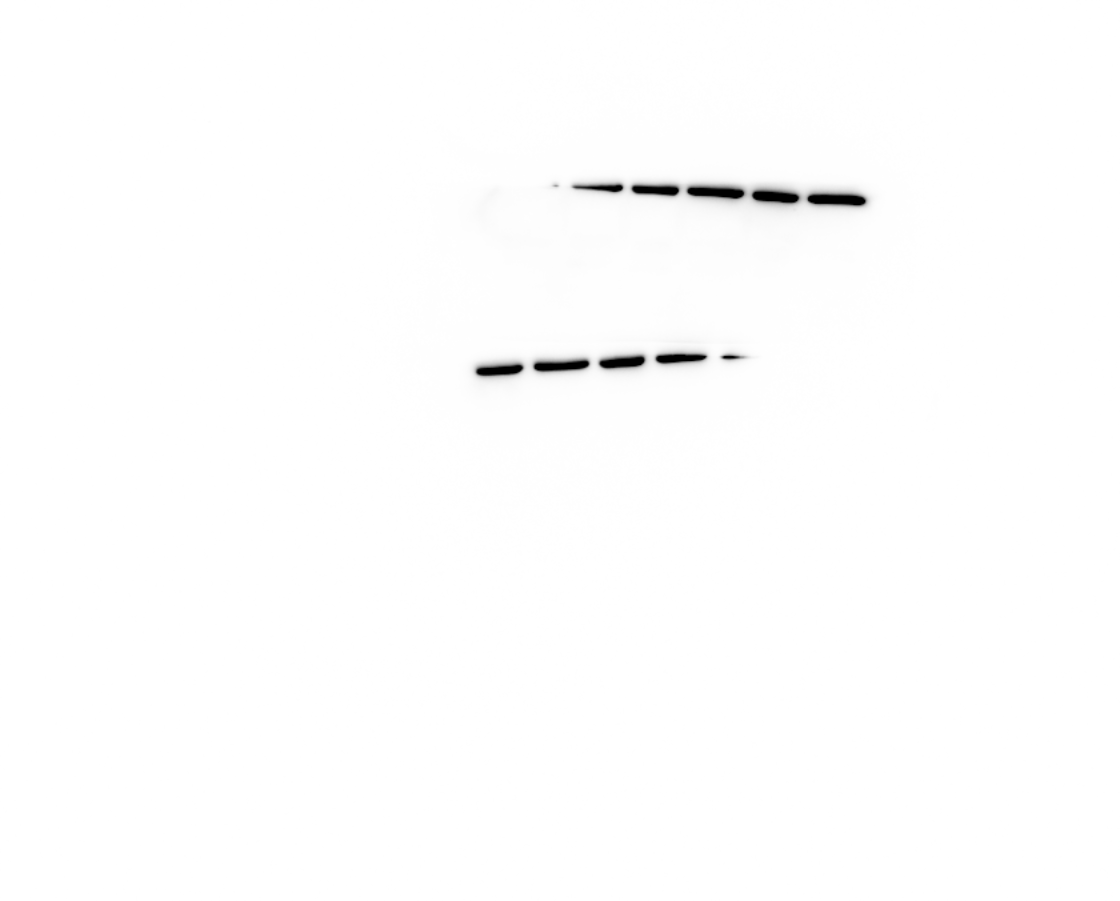

Supplement: Figure 5—figure supplement 1—source data 1. [file elife-69219-fig5-figsupp1-data1.zip › Figure 5-WesternBlot-source data/Figure 5-WesternBlot-source data/Fig.5.D.3-BetaActinx2_raw.tif]

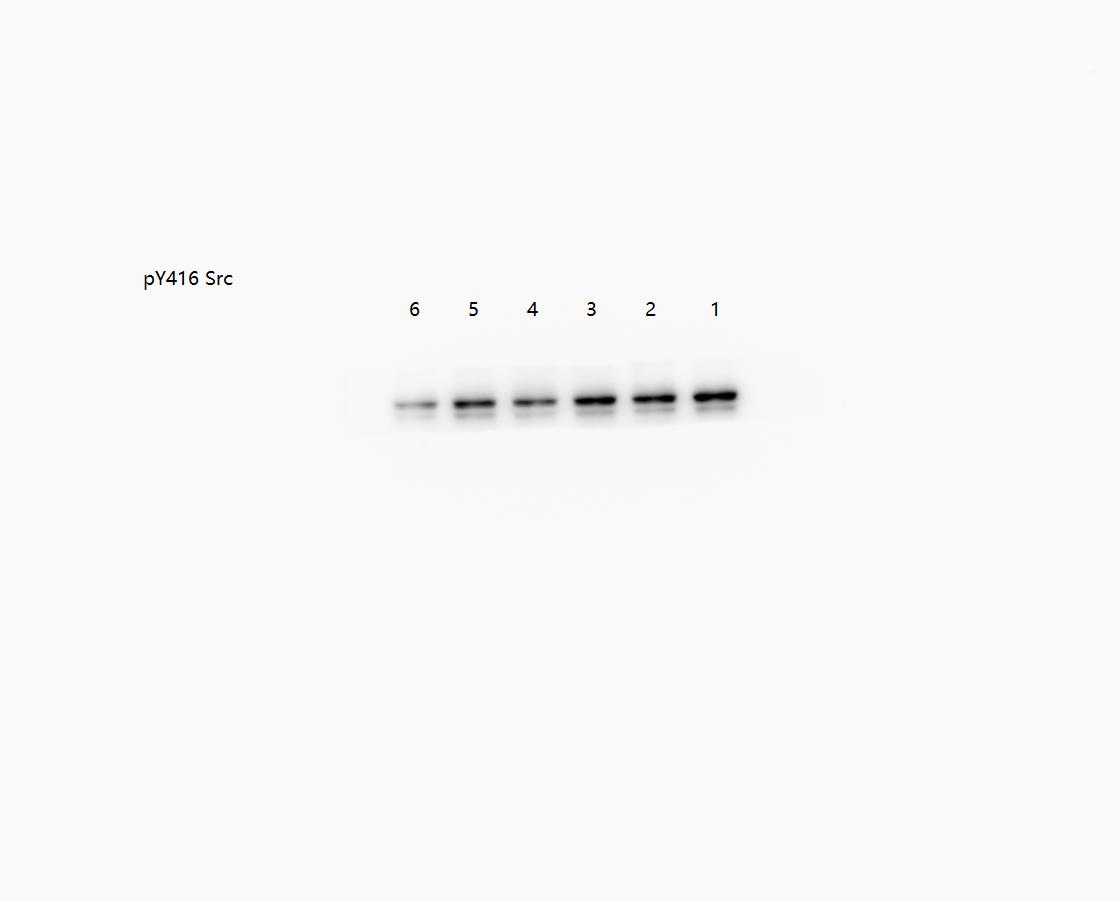

Supplement: Figure 5—figure supplement 1—source data 1. [file elife-69219-fig5-figsupp1-data1.zip › Figure 5-WesternBlot-source data/Figure 5-WesternBlot-source data/Fig.5.D.4.pY416-Src_labeled.TIF]

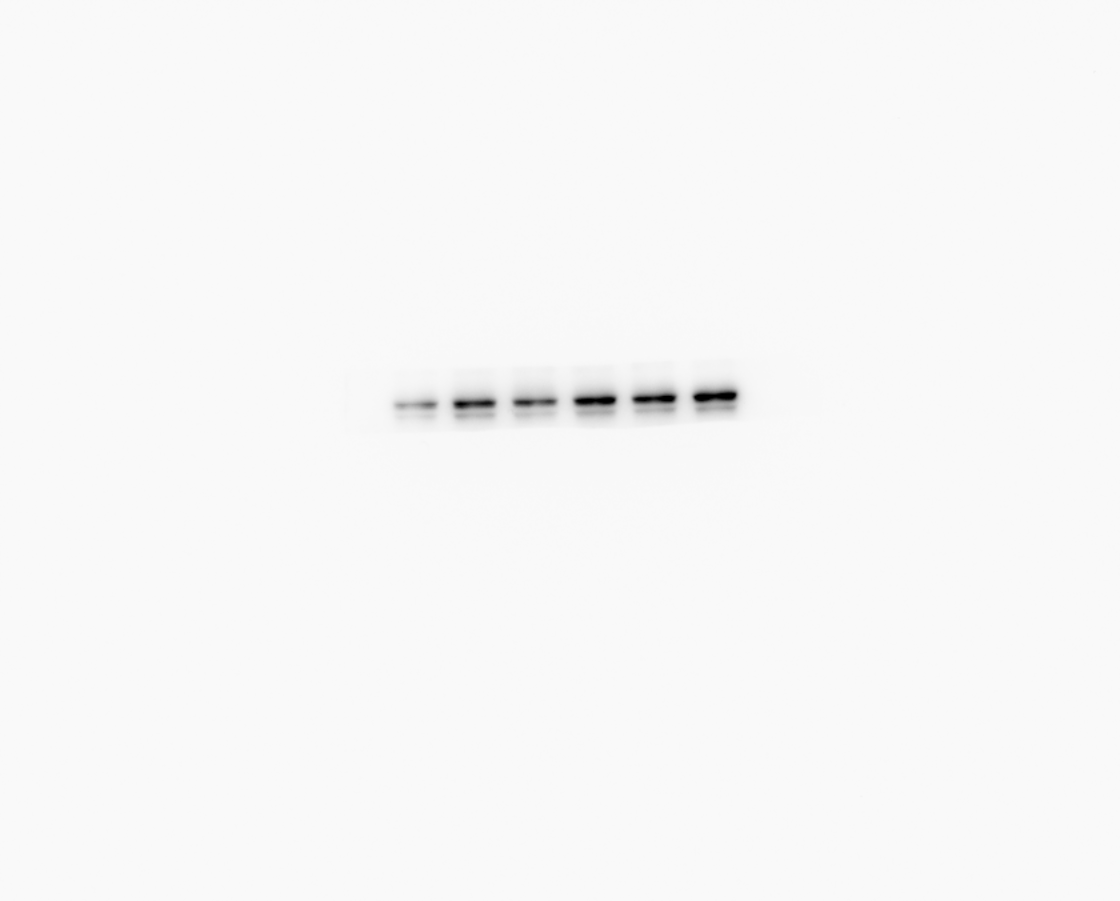

Supplement: Figure 5—figure supplement 1—source data 1. [file elife-69219-fig5-figsupp1-data1.zip › Figure 5-WesternBlot-source data/Figure 5-WesternBlot-source data/Fig.5.D.4.pY416-Src_raw.tif]

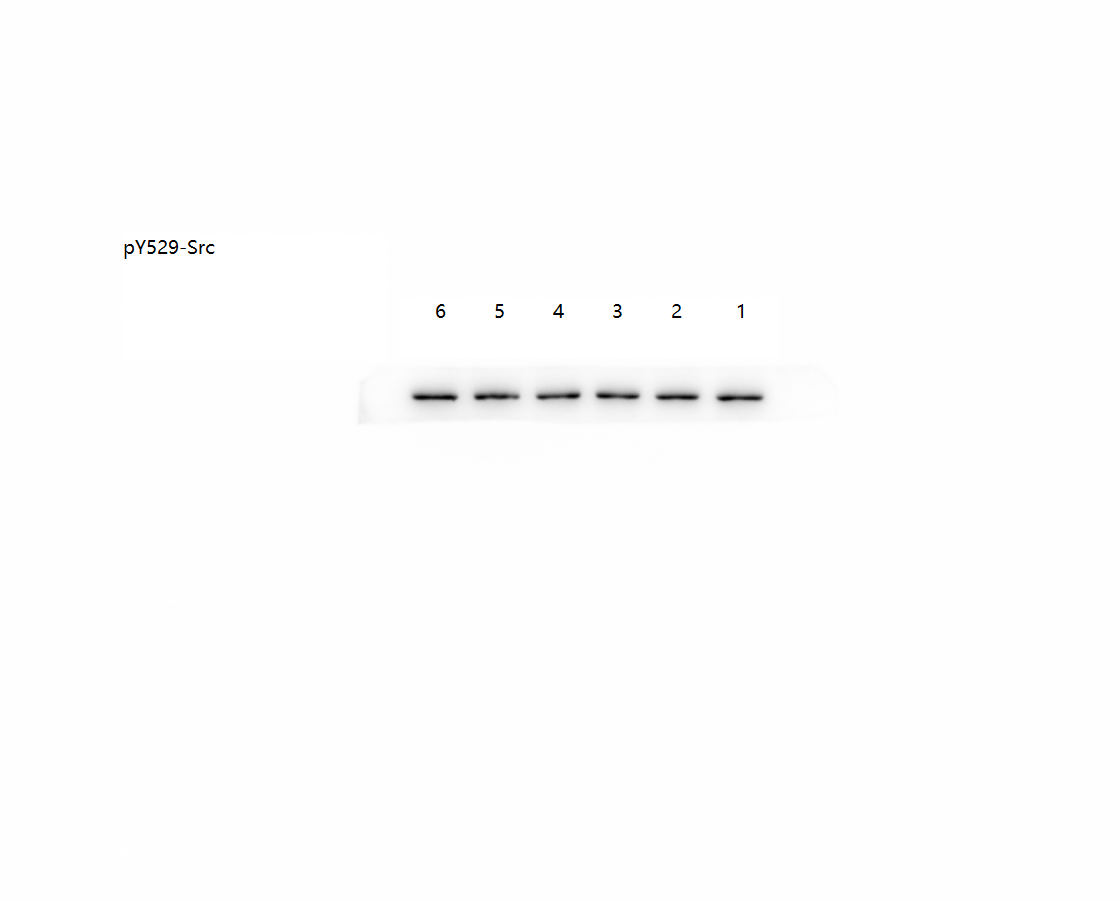

Supplement: Figure 5—figure supplement 1—source data 1. [file elife-69219-fig5-figsupp1-data1.zip › Figure 5-WesternBlot-source data/Figure 5-WesternBlot-source data/Fig.5.D.5.pY529-Src_labeled.TIF]

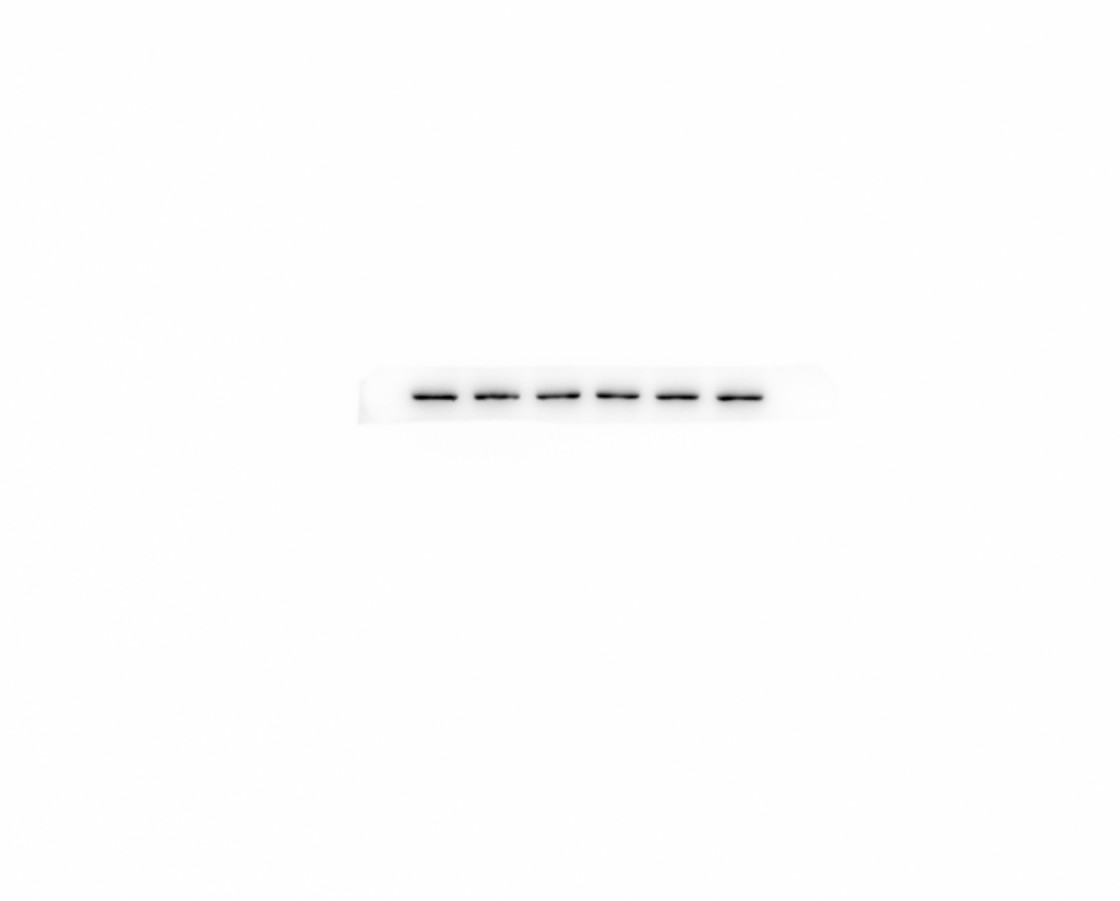

Supplement: Figure 5—figure supplement 1—source data 1. [file elife-69219-fig5-figsupp1-data1.zip › Figure 5-WesternBlot-source data/Figure 5-WesternBlot-source data/Fig.5.D.5.pY529-Src_raw.tif]

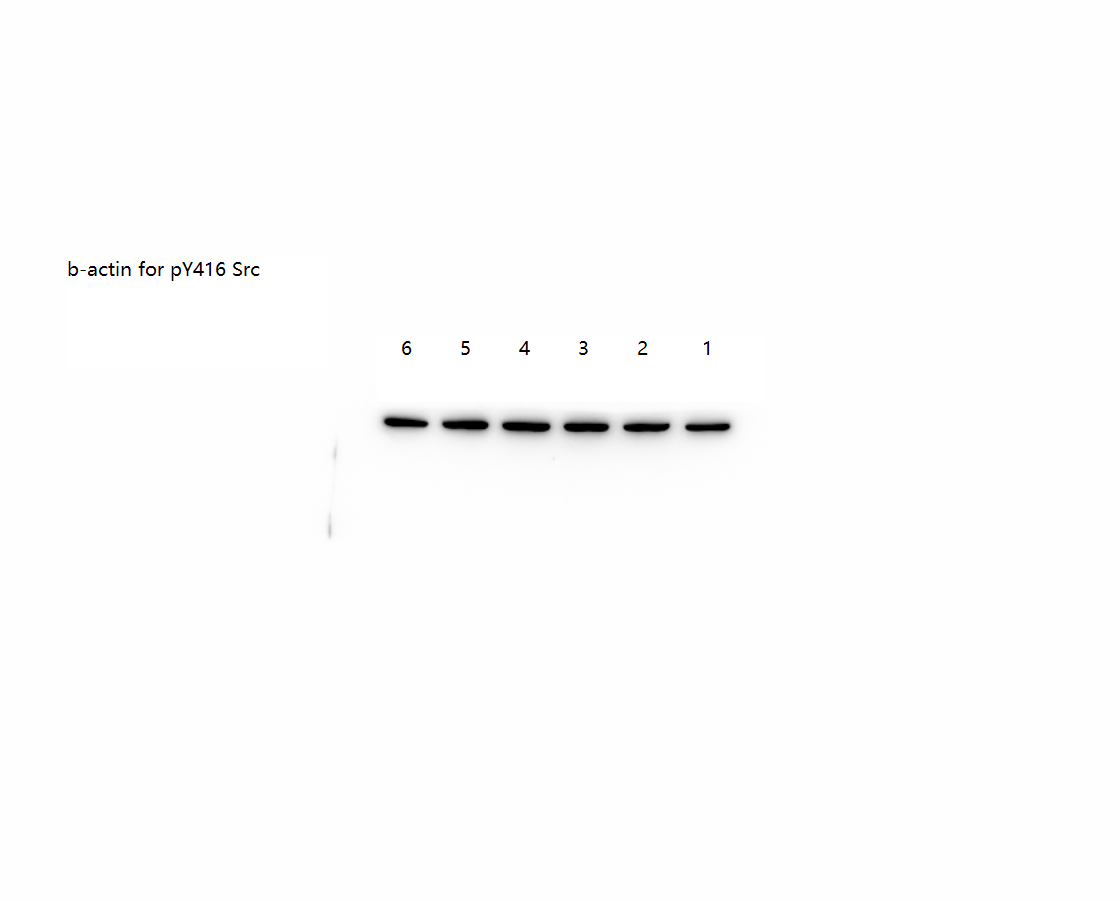

Supplement: Figure 5—figure supplement 1—source data 1. [file elife-69219-fig5-figsupp1-data1.zip › Figure 5-WesternBlot-source data/Figure 5-WesternBlot-source data/Fig.5.D.6.BetaActin for pY416-Src_labeled.TIF]

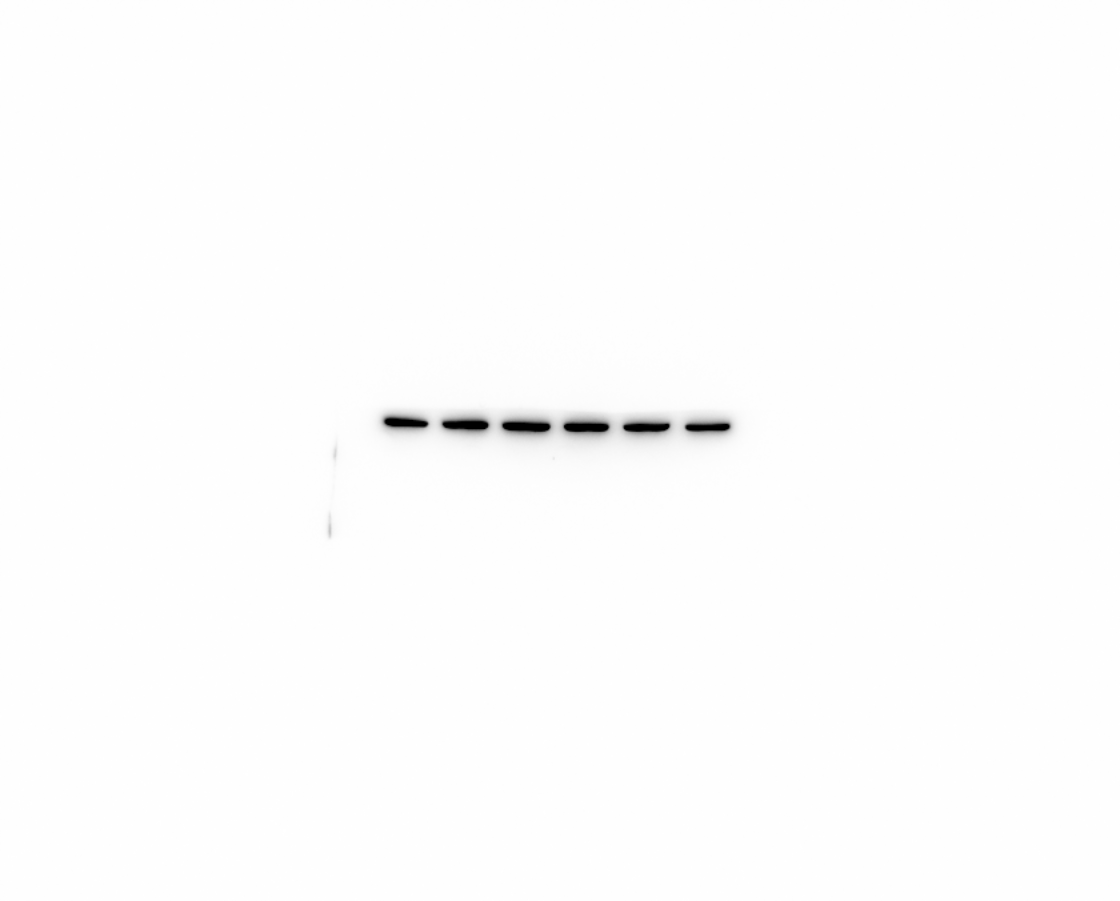

Supplement: Figure 5—figure supplement 1—source data 1. [file elife-69219-fig5-figsupp1-data1.zip › Figure 5-WesternBlot-source data/Figure 5-WesternBlot-source data/Fig.5.D.6.BetaActin for pY416-Src_raw.tif]

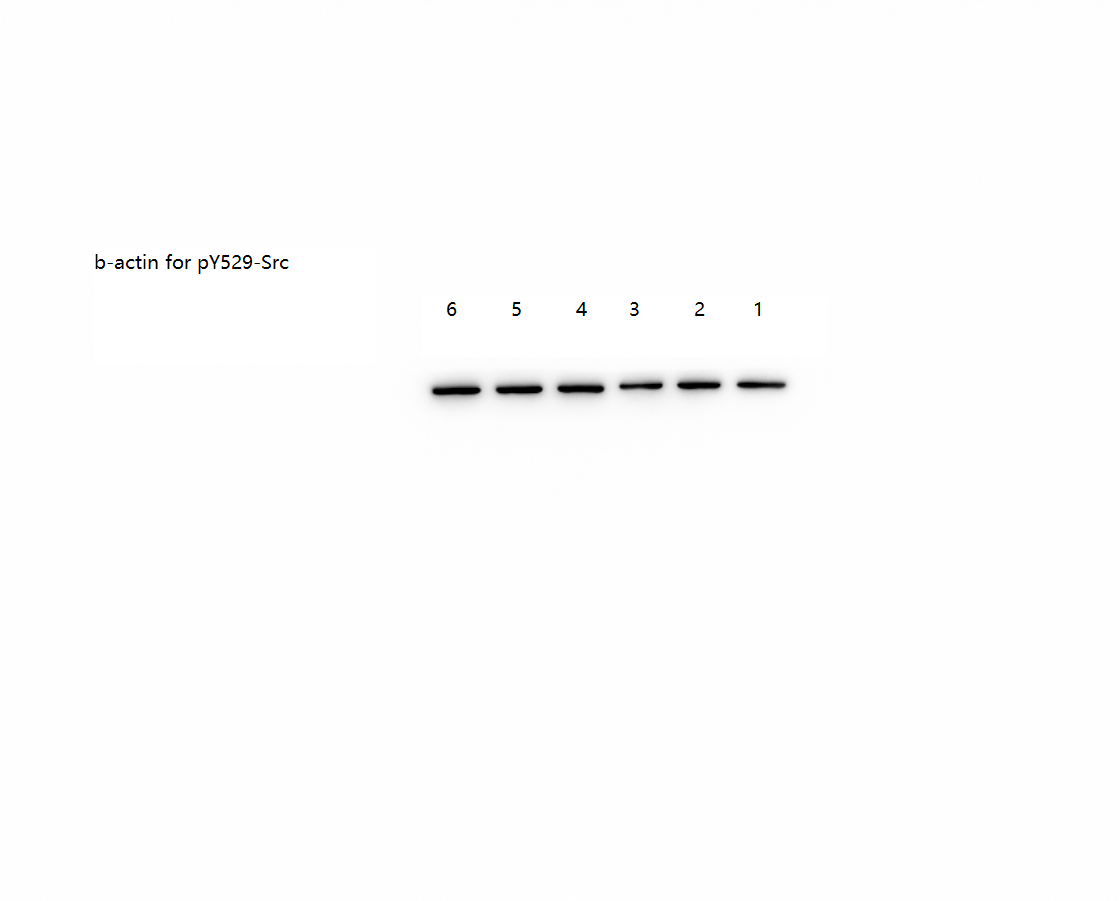

Supplement: Figure 5—figure supplement 1—source data 1. [file elife-69219-fig5-figsupp1-data1.zip › Figure 5-WesternBlot-source data/Figure 5-WesternBlot-source data/Fig.5.D.7.BetaActin for pY529-Src_labeled.TIF]

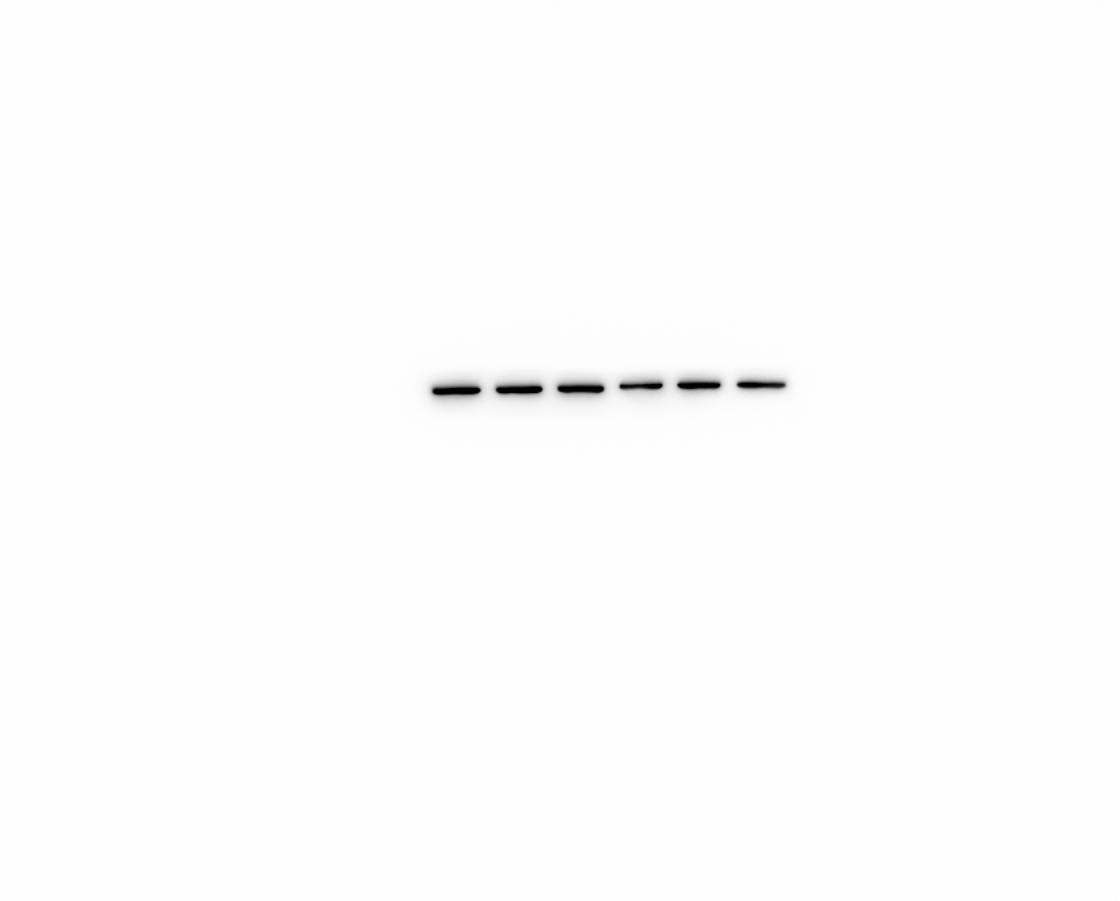

Supplement: Figure 5—figure supplement 1—source data 1. [file elife-69219-fig5-figsupp1-data1.zip › Figure 5-WesternBlot-source data/Figure 5-WesternBlot-source data/Fig.5.D.7.BetaActin for pY529-Src_raw.tif]

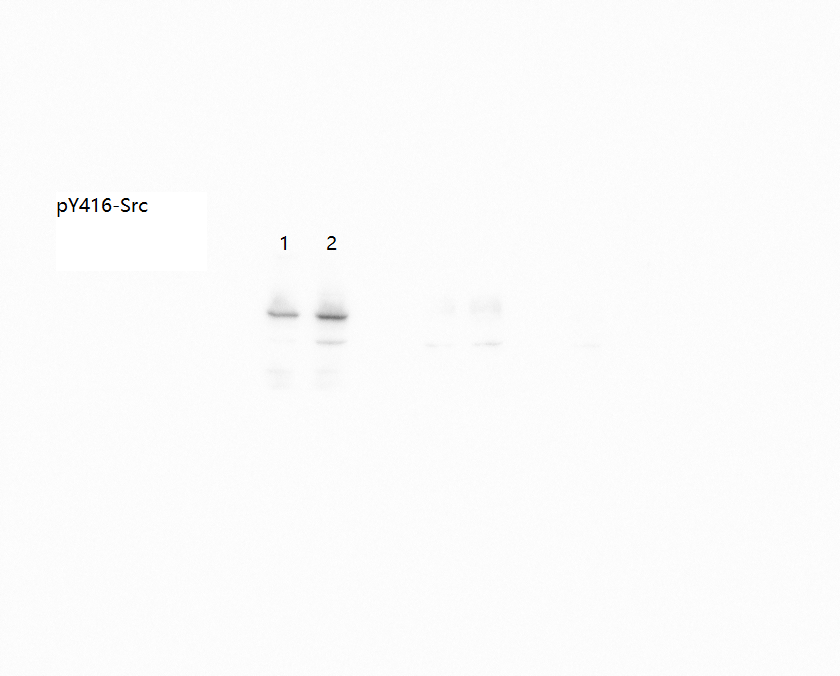

Supplement: Figure 5—figure supplement 1—source data 1. [file elife-69219-fig5-figsupp1-data1.zip › Figure 5-WesternBlot-source data/Figure 5-WesternBlot-source data/Fig.5.G.1.pY416-Src_labeled.TIF]

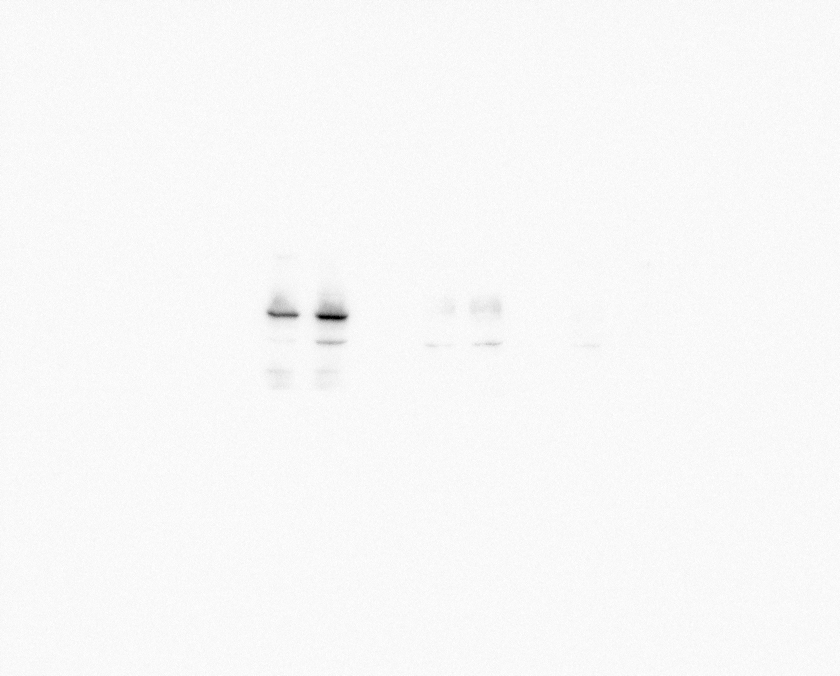

Supplement: Figure 5—figure supplement 1—source data 1. [file elife-69219-fig5-figsupp1-data1.zip › Figure 5-WesternBlot-source data/Figure 5-WesternBlot-source data/Fig.5.G.1.pY416-Src_raw.tif]

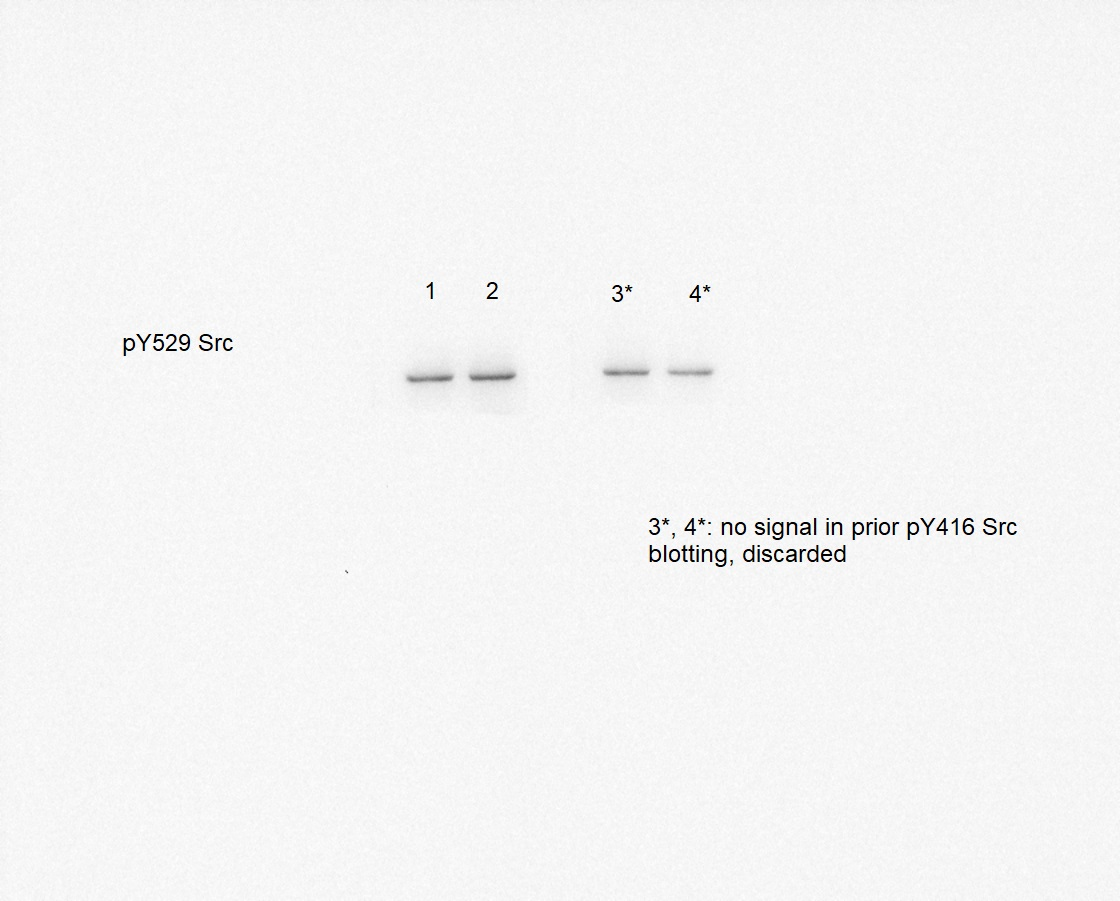

Supplement: Figure 5—figure supplement 1—source data 1. [file elife-69219-fig5-figsupp1-data1.zip › Figure 5-WesternBlot-source data/Figure 5-WesternBlot-source data/Fig.5.G.2.pY529-Src_labeled.TIF]

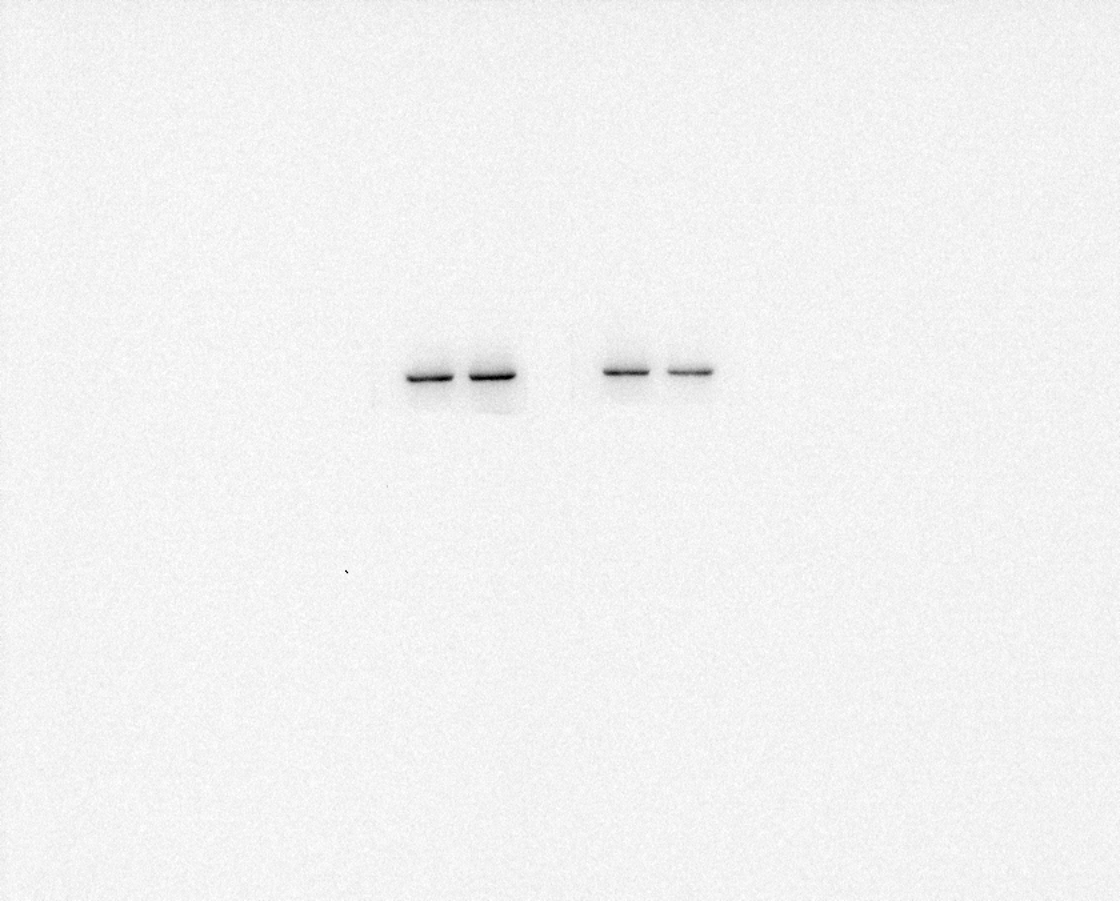

Supplement: Figure 5—figure supplement 1—source data 1. [file elife-69219-fig5-figsupp1-data1.zip › Figure 5-WesternBlot-source data/Figure 5-WesternBlot-source data/Fig.5.G.2.pY529-Src_raw.tif]

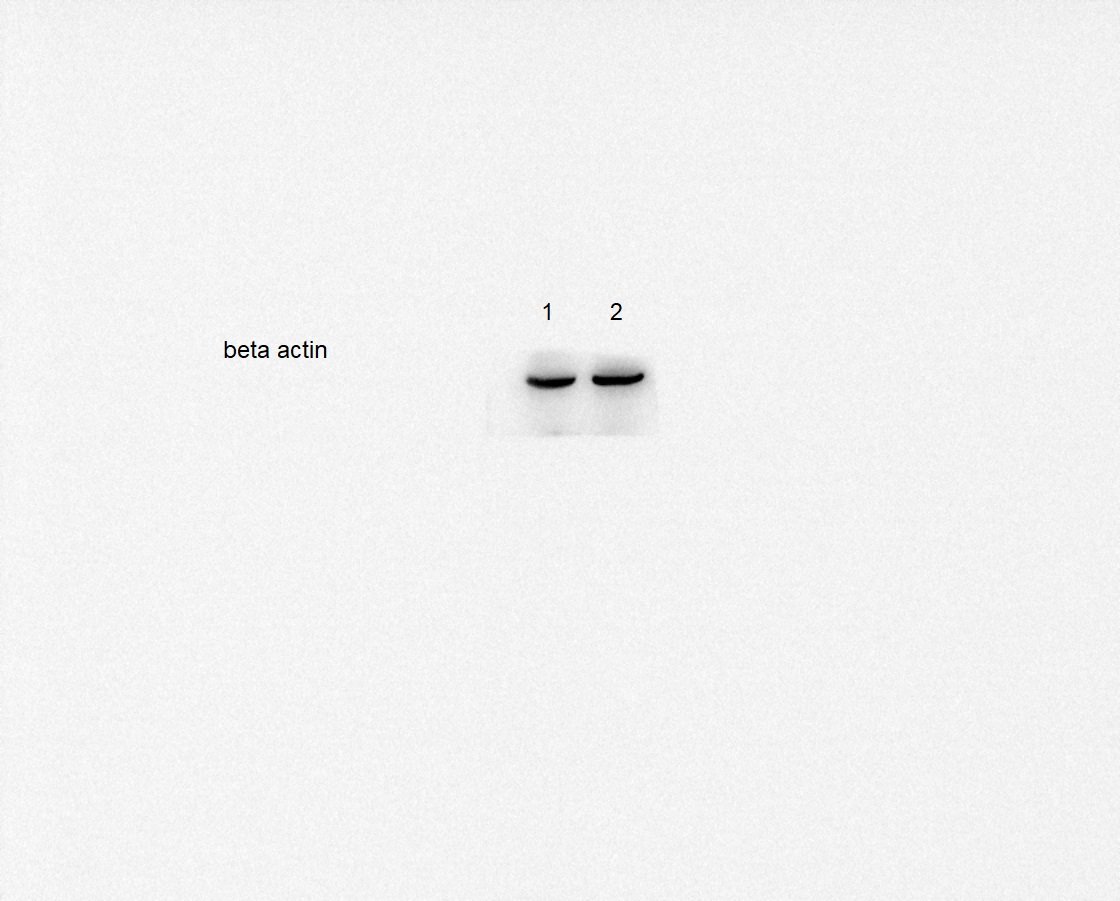

Supplement: Figure 5—figure supplement 1—source data 1. [file elife-69219-fig5-figsupp1-data1.zip › Figure 5-WesternBlot-source data/Figure 5-WesternBlot-source data/Fig.5.G.3.BetaActin_labeled.TIF]

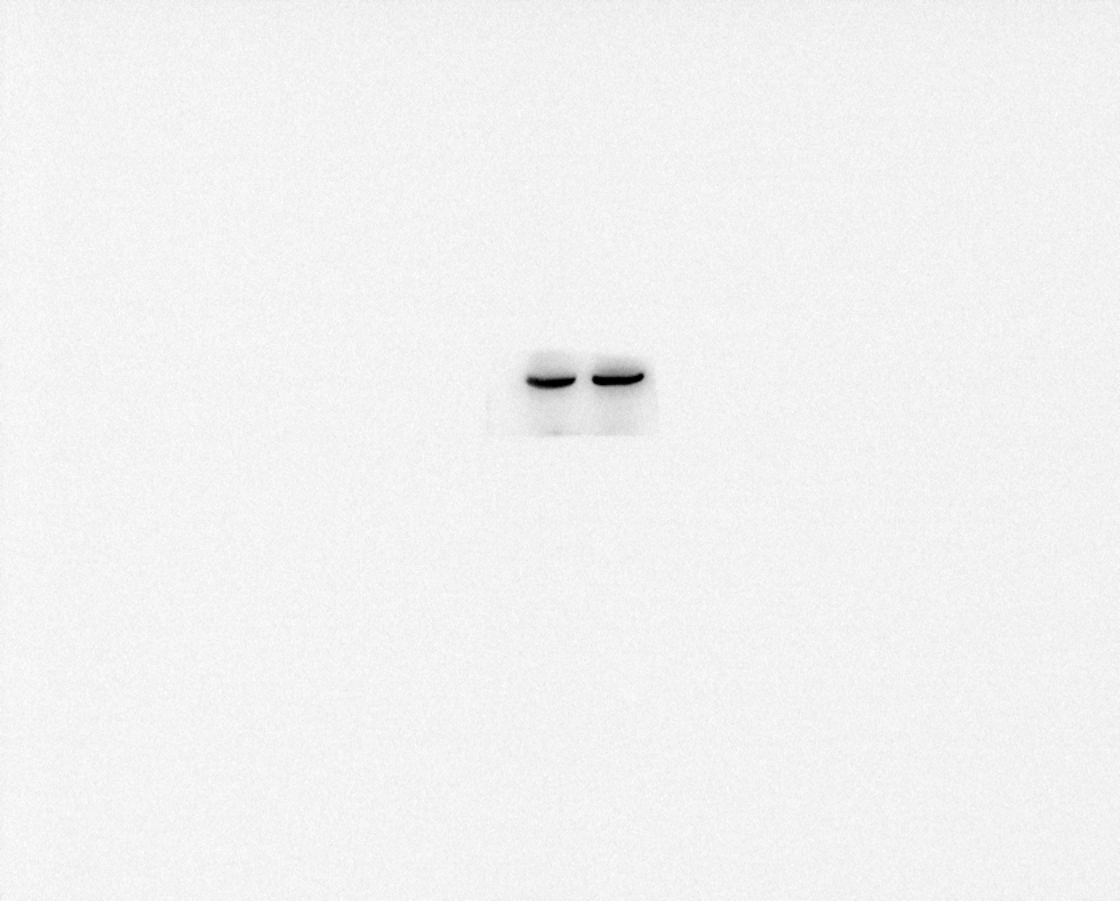

Supplement: Figure 5—figure supplement 1—source data 1. [file elife-69219-fig5-figsupp1-data1.zip › Figure 5-WesternBlot-source data/Figure 5-WesternBlot-source data/Fig.5.G.3.BetaActin_raw.tif]

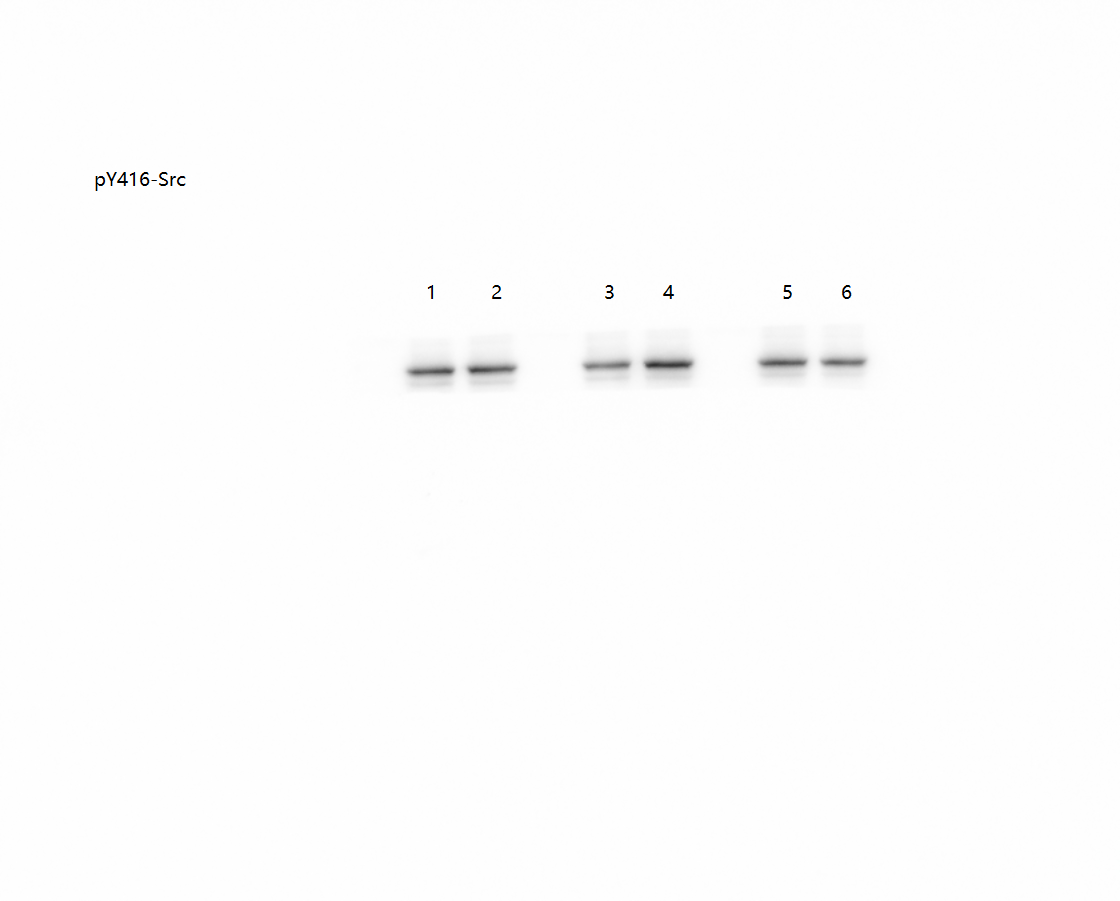

Supplement: Figure 5—figure supplement 1—source data 1. [file elife-69219-fig5-figsupp1-data1.zip › Figure 5-WesternBlot-source data/Figure 5-WesternBlot-source data/Fig.5.G.4.pY416-Src_labeled.tif]

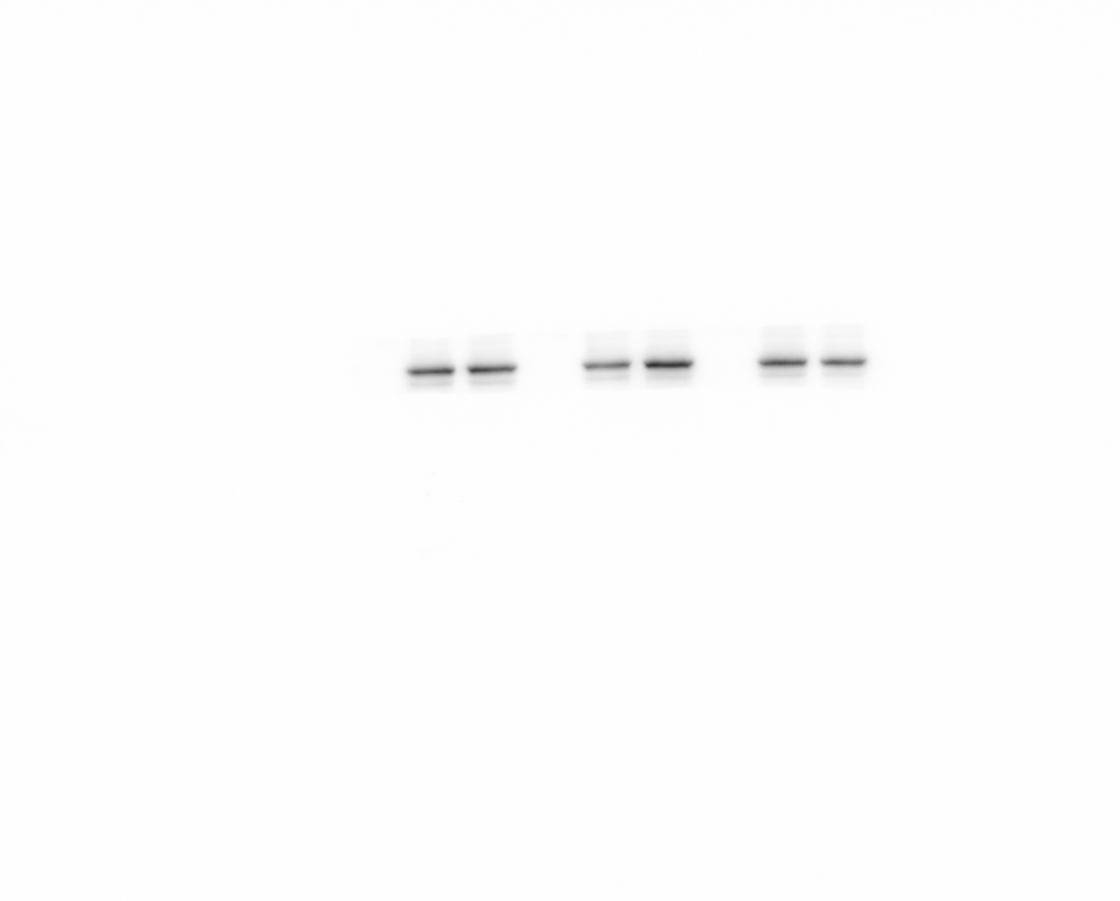

Supplement: Figure 5—figure supplement 1—source data 1. [file elife-69219-fig5-figsupp1-data1.zip › Figure 5-WesternBlot-source data/Figure 5-WesternBlot-source data/Fig.5.G.4.pY416-Src_raw.tif]

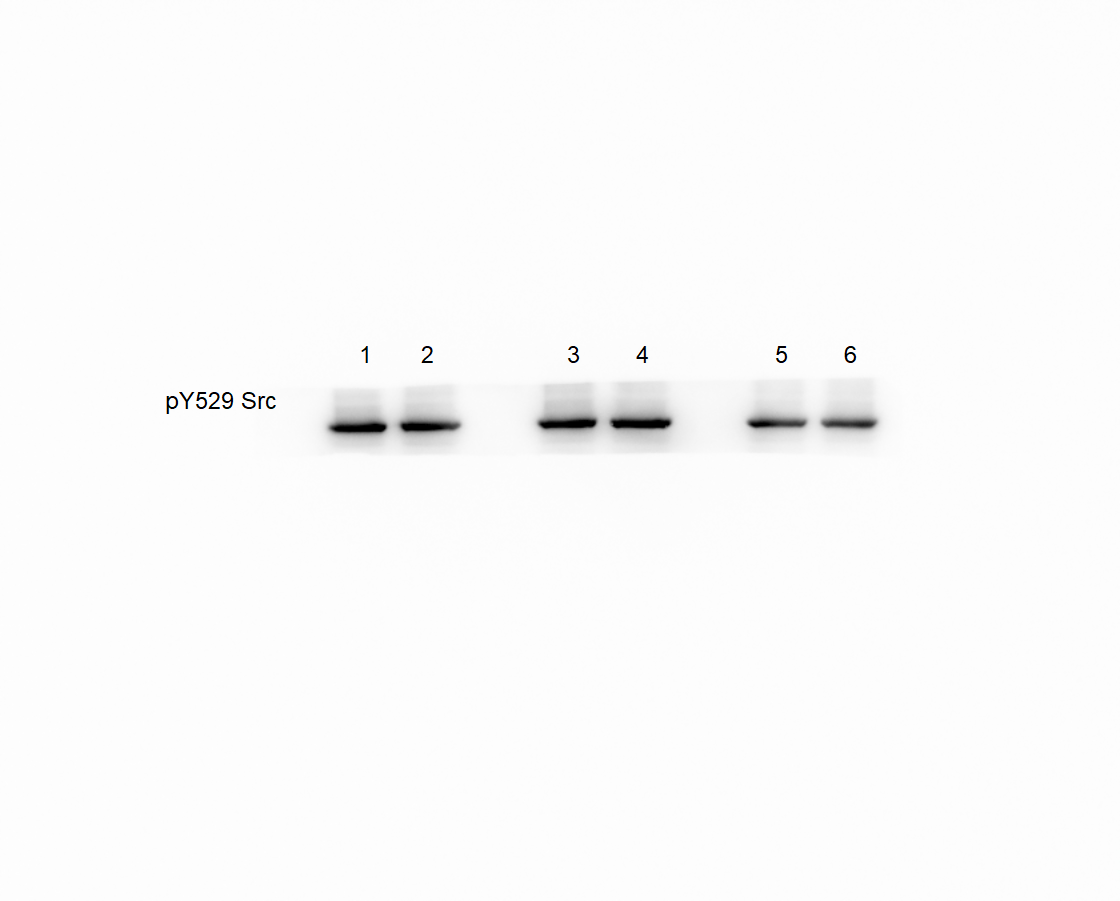

Supplement: Figure 5—figure supplement 1—source data 1. [file elife-69219-fig5-figsupp1-data1.zip › Figure 5-WesternBlot-source data/Figure 5-WesternBlot-source data/Fig.5.G.5.pY529-Src_labeled.tif]

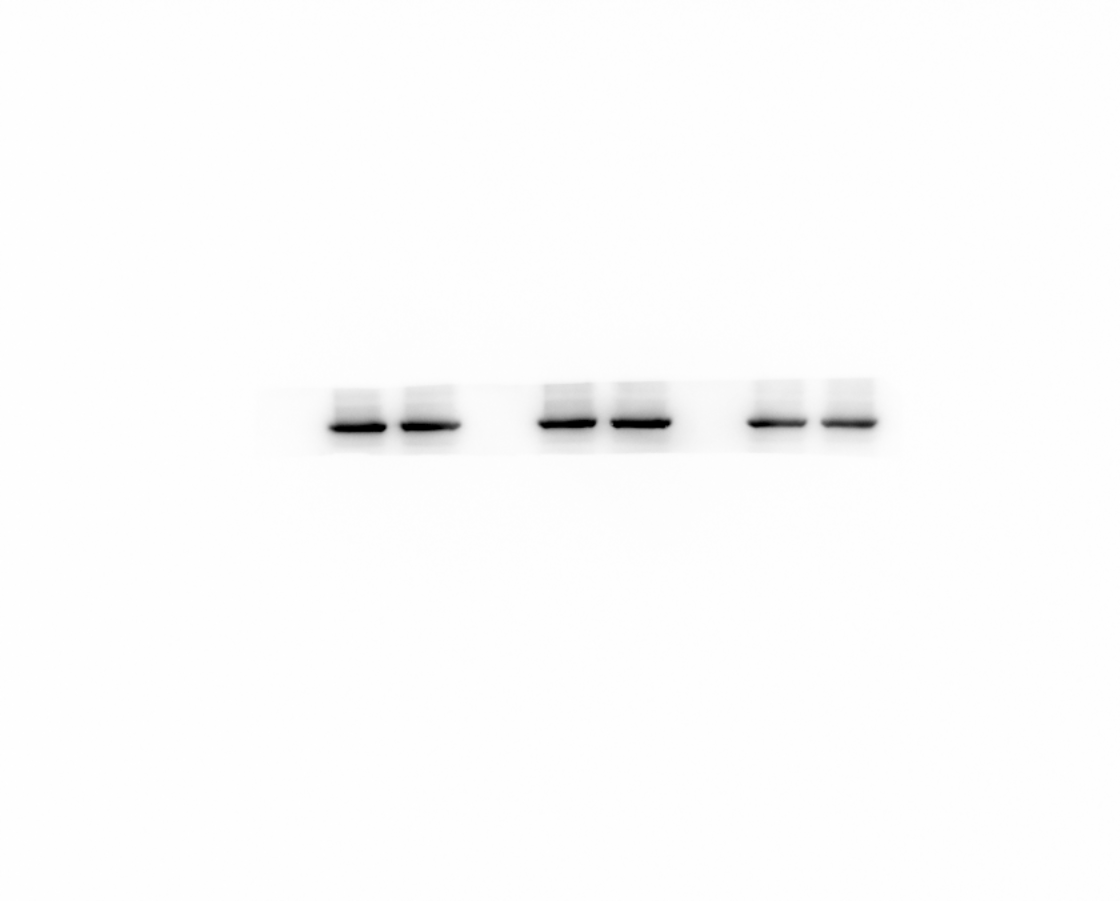

Supplement: Figure 5—figure supplement 1—source data 1. [file elife-69219-fig5-figsupp1-data1.zip › Figure 5-WesternBlot-source data/Figure 5-WesternBlot-source data/Fig.5.G.5.pY529-Src_raw.tif]

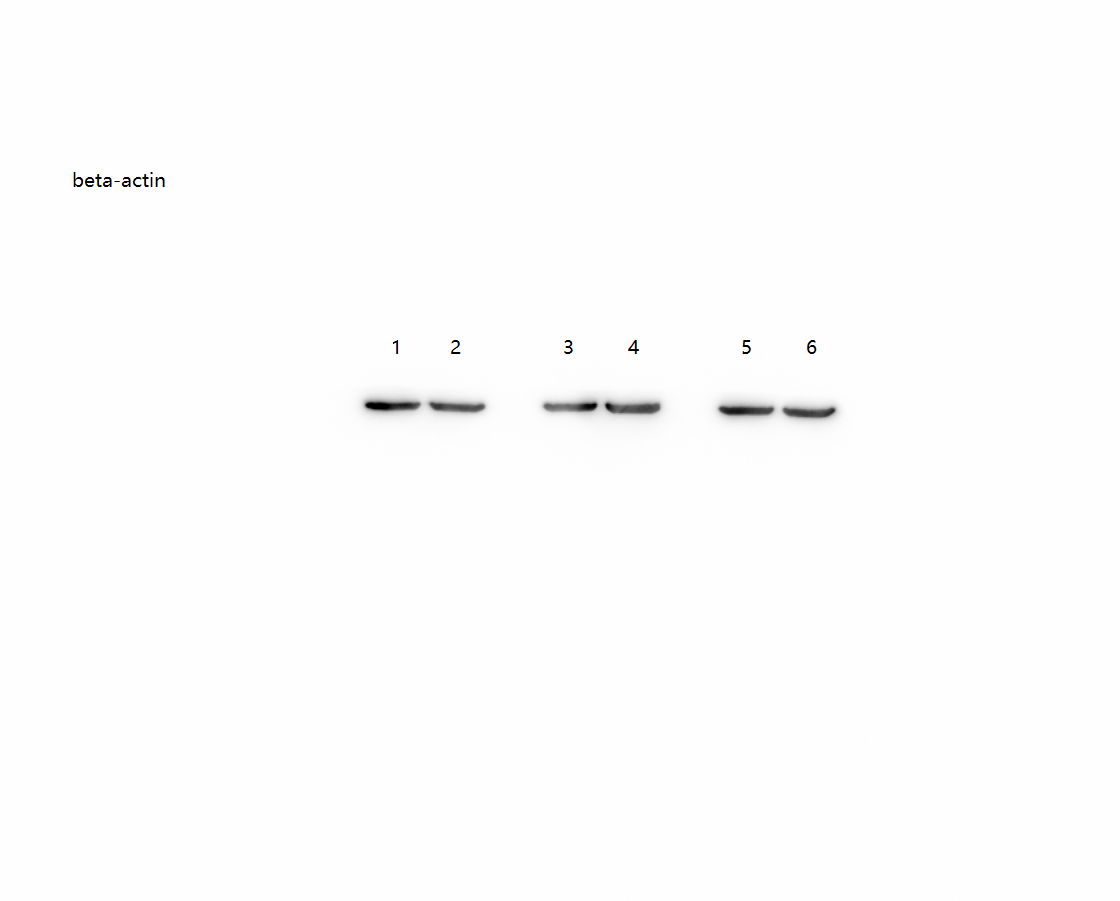

Supplement: Figure 5—figure supplement 1—source data 1. [file elife-69219-fig5-figsupp1-data1.zip › Figure 5-WesternBlot-source data/Figure 5-WesternBlot-source data/Fig.5.G.6.BetaActin_labeled.tif]

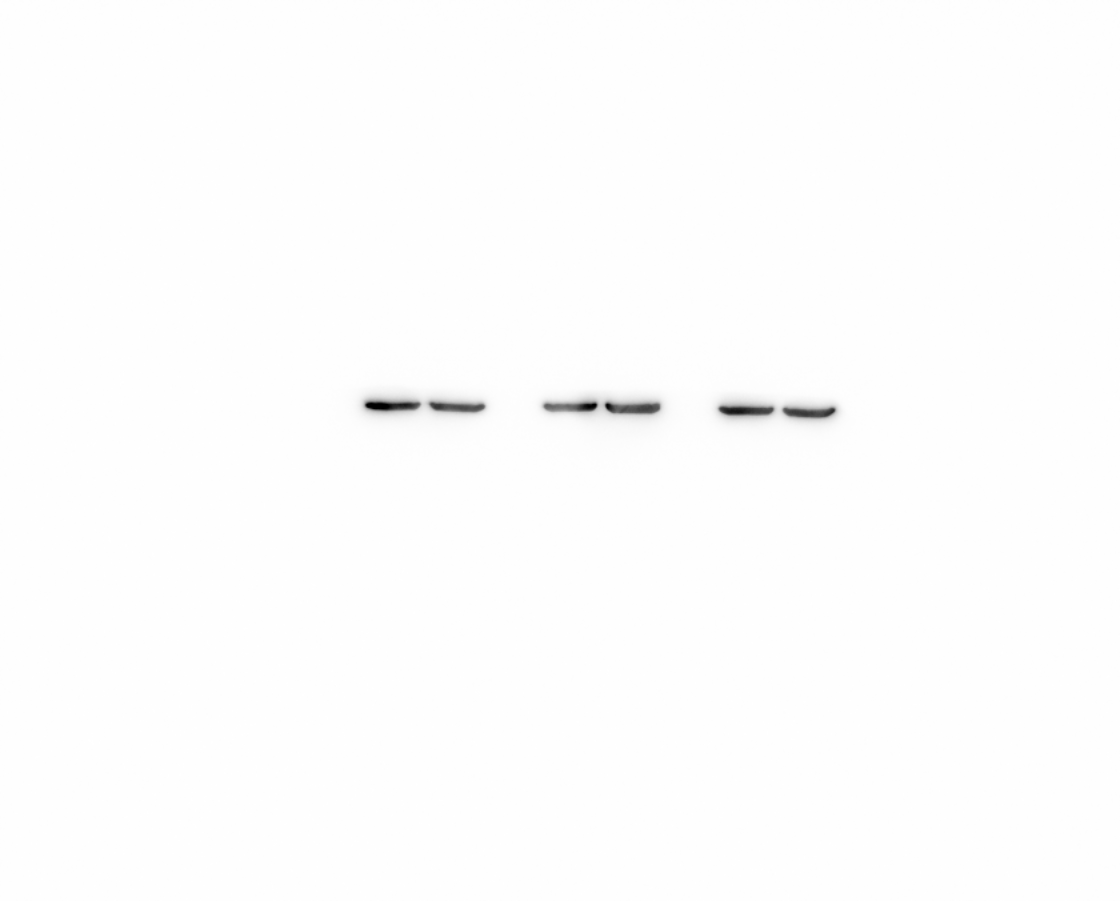

Supplement: Figure 5—figure supplement 1—source data 1. [file elife-69219-fig5-figsupp1-data1.zip › Figure 5-WesternBlot-source data/Figure 5-WesternBlot-source data/Fig.5.G.6.BetaActin_raw.tif]
