# Supplementary material for: Endothelial SIRPα signaling controls VE-cadherin endocytosis for thymic homing of progenitor cells
Source: eLife. 2022 May 5;11:e69219. doi: 10.7554/eLife.69219 (PMC9071265; doi:10.7554/eLife.69219)
Supplement: Transparent reporting form [file elife-69219-transrepform1.docx]

***eLife’s* transparent reporting form**

We encourage authors to provide detailed information *within their submission* to facilitate the interpretation and replication of experiments. Authors can upload supporting documentation to indicate the use of appropriate reporting guidelines for health-related research (see [EQUATOR Network](http://www.equator-network.org/%20)), life science research (see the [BioSharing Information Resource](https://biosharing.org/)), or the [ARRIVE guidelines](http://www.plosbiology.org/article/info:doi/10.1371/journal.pbio.1000412) for reporting work involving animal research. Where applicable, authors should refer to any relevant reporting standards documents in this form.

If you have any questions, please consult our Journal Policies and/or contact us: [editorial@elifesciences.org](mailto:editorial@elifesciences.org).

**Sample-size estimation**

- You should state whether an appropriate sample size was computed when the study was being designed
- You should state the statistical method of sample size computation and any required assumptions
- If no explicit power analysis was used, you should describe how you decided what sample (replicate) size (number) to use

Please outline where this information can be found within the submission (e.g., sections or figure legends), or explain why this information doesn’t apply to your submission:

The sample size for each experimental group is indicated in the figure legends. We did not predetermine sample size by calculation. The exact n values used to calculate the statistics are provided in the results and a reasonable sample size was chosen to ensure adequate reproducibility of results.

**Replicates**

- You should report how often each experiment was performed
- You should include a definition of biological versus technical replication
- The data obtained should be provided and sufficient information should be provided to indicate the number of independent biological and/or technical replicates
- If you encountered any outliers, you should describe how these were handled
- Criteria for exclusion/inclusion of data should be clearly stated
- High-throughput sequence data should be uploaded before submission, with a private link for reviewers provided (these are available from both GEO and ArrayExpress)

Please outline where this information can be found within the submission (e.g., sections or figure legends), or explain why this information doesn’t apply to your submission:

All experiments have been performed independently for at least two times. The specific information is shown in the figure legends for each experiment. For outlier exclusion, Grubbs'test (extreme studentized deviate, from [Graphpad outlier calculator](https://www.graphpad.com/quickcalcs/Grubbs1.cfm)) is used to determine whether one of the values is a significant outlier from the rest (alpha=0.01), after outlier exclusion, sample size should meet the requirement.

**Statistical reporting**

- Statistical analysis methods should be described and justified
- Raw data should be presented in figures whenever informative to do so (typically when N per group is less than 10)
- For each experiment, you should identify the statistical tests used, exact values of N, definitions of center, methods of multiple test correction, and dispersion and precision measures (e.g., mean, median, SD, SEM, confidence intervals; and, for the major substantive results, a measure of effect size (e.g., Pearson's r, Cohen's d)
- Report exact p-values wherever possible alongside the summary statistics and 95% confidence intervals. These should be reported for all key questions and not only when the p-value is less than 0.05.

Please outline where this information can be found within the submission (e.g., sections or figure legends), or explain why this information doesn’t apply to your submission:

Meaning of error bars, methods for significance test and significance levels can be found within figure legends and Statistical Analysis part in method section.

Raw data are presented in most of figures except:

1) In transmigration assay, raw value (transmigrated lymphocytes) of each sample is normalized to (divided by) the average transmigrated lymphocytes of control group (wildtype, empty vector, etc), so that mean value of control group equals to 1. Raw data are available in supplementary file 1.

2) In quantification of western blotting, raw values (grayscale of chemiluminescence) of target protein in each independent assay are firstly normalized to its corresponding beta-actin control. After that, each sample (beta-actin normalized) is normalized to the average of control group, so that mean value of control group equals to 1. Raw data are available in supplementary file 1.

(For large datasets, or papers with a very large number of statistical tests, you may upload a single table file with tests, Ns, etc., with reference to sections in the manuscript.)

**Group allocation**

- Indicate how samples were allocated into experimental groups (in the case of clinical studies, please specify allocation to treatment method); if randomization was used, please also state if restricted randomization was applied
- Indicate if masking was used during group allocation, data collection and/or data analysis

Please outline where this information can be found within the submission (e.g., sections or figure legends), or explain why this information doesn’t apply to your submission:

Mice were randomized into different groups. Masking was not performed due to the unambiguous nature of measurements and systematic analyses used in these experiments.

**Additional data files (“source data”)**

- We encourage you to upload relevant additional data files, such as numerical data that are represented as a graph in a figure, or as a summary table
- Where provided, these should be in the most useful format, and they can be uploaded as “Source data” files linked to a main figure or table
- Include model definition files including the full list of parameters used
- Include code used for data analysis (e.g., R, MatLab)
- Avoid stating that data files are “available upon request”

Please indicate the figures or tables for which source data files have been provided:

All data generated or analyzed during this study are included in the manuscript, supplementary file 1, and listed reference.
